# Supplementary material for: A Coating Based on Bioactive Compounds from Streptomyces spp. and Chitosan Oligomers to Control Botrytis cinerea Preserves the Quality and Improves the Shelf Life of Table Grapes
Source: Plants (Basel). 2023 Jan 28;12(3):577. doi: 10.3390/plants12030577 (PMC9921524; doi:10.3390/plants12030577)
Supplement: Supplementary file 1 [file plants-12-00577-s001.zip › plants-2138792-supplementary.pdf]

A Coating based on Bioactive Compounds from *Streptomyces* spp. and Chitosan Oligomers to Control *Botrytis cinerea,* Preserve the Quality and Improve the Shelf Life of Table Grapes

Laura Buzón-Durán, Eva Sánchez-Hernández, Mercedes Sánchez-Báscones, Mari Cruz García-González, Salvador Hernández-Navarro, Adriana Correa-Guimarães and Pablo Martín-Ramos

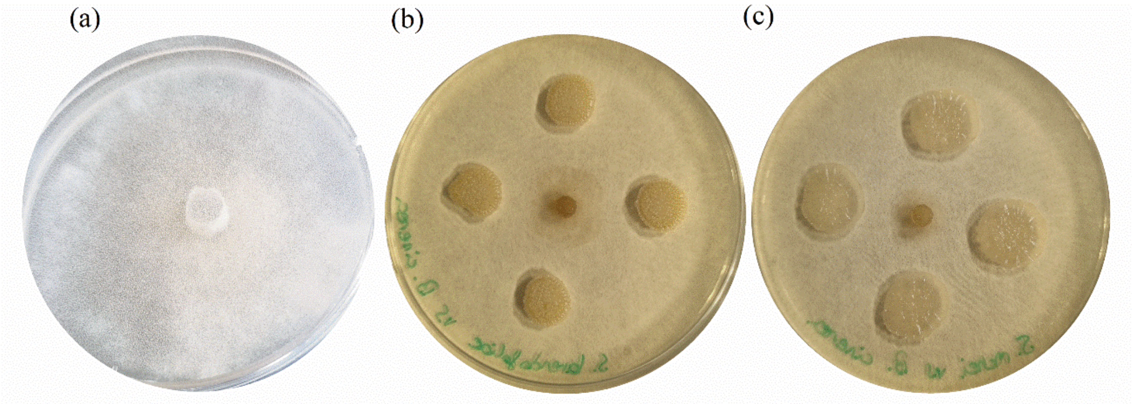

Figure S1. Antagonism of *Streptomyces* spp. against *B. cinerea* in dual plate assays: (**a**) PDA control; (**b**) *S. lavendofoliae* DSM 40217 versus *B. cinerea*; (**c**) *S. rochei* DSM 41729 versus *B. cinerea*. Only one replicate per treatment is shown.

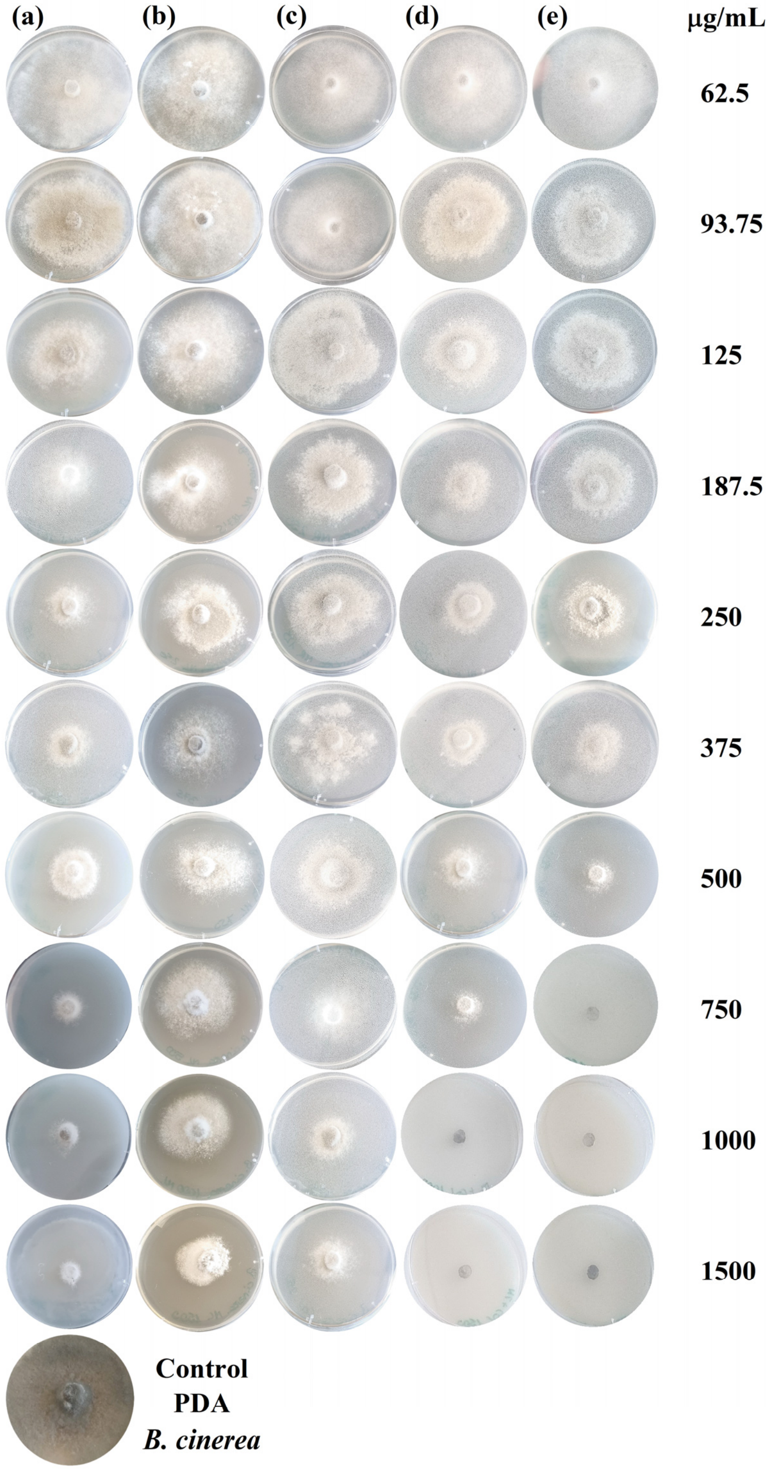

Figure S2. Results of *B. cinerea* mycelial growth inhibition assays: (**a**) chitosan oligomers; (**b**) *S. lavendofoliae* DSM 40217 secondary metabolites; (**c**) *S. rochei* DSM 41729 secondary metabolites; (**d**) COS-*S. lavendofoliae* DSM 40217 secondary metabolites; (**e**) COS-*S. rochei* DSM 41729 secondary metabolites. The concentration of the treatments decreases from top to bottom. The Petri dish in the bottom left corner shows the PDA control. Only one replicate per treatment and dose is shown.

Table S1. Chemical species identified by GC-MS in *S. lavendofoliae* DSM 40217 extract.

RT (min)	Area (%)	Assignment
3.3600	0.125	Butanoic acid, 3-hydroxy-, ethyl ester
3.4371	0.236	Butanamide, 3-methyl-
3.5202	0.110	1,2-Propanediol, 3-methoxy-
4.3512	0.163	Methional
4.4580	0.144	Pyrazine, 2,5-dimethyl-
4.5233	0.139	1,2,4-Cyclopentanetrione, 3,3-dimethyl-
4.9091	0.425	Butyrolactone
4.9625	0.661	1,4-Dioxaspiro[2.4]heptan-5-one, 7-methyl-
5.0278	0.556	1,2-Cyclopentanedione
5.2711	0.451	2-Methylpyrazolo[3,4-d]tetrazolo[4,5-b]pyrimidin-4(2H)-one
5.7697	1.590	3(5)-[[1,2-Dihydroxy-3-propoxy]methyl]-4-hydroxy-1H-pyrazole-5(3)-carboxamide
5.9062	3.438	Borane, compd. with dimethylamine (1:1)
6.0546	1.359	N,N-Dimethylglycine
6.1496	0.516	Piperazine, 2,5-dimethyl-, trans-
6.1970	0.250	Tetramethylammonium perchlorate
6.2445	0.990	N,N-Dimethylglycine
6.4285	1.341	Benzeneacetaldehyde
6.4938	3.962	Benzeneacetaldehyde
6.6837	0.689	1H-Pyrazole, 3-methyl-
6.8084	0.314	9-Azabicyclo[6.1.0]non-4-en-9-amine, (1.alpha.,4Z,8.alpha.)-
6.8499	0.542	N-Methyl-N-(N,N-dimethylaminoethyl)-aminoethanol
6.9924	0.662	2-Pyrrolidinone
7.0814	0.331	3,7-Dihydropurine-2,6-dione, 7-(2-dimethylaminoethyl)-3-methyl-
7.2832	0.445	N,N',N''-Trimethyldiitrimethylenetriamine
7.3248	0.298	2,5-Dihydro-5-methoxy-2-furanone
7.4078	0.823	1,3-Propanediamine, N-methyl-
7.5088	0.267	5,6-Diamino-1,3-dimethyluracil
7.5622	0.210	3-Piperidinol, 1,4-dimethyl-, trans-
7.6749	0.242	4,5-Diamino-6-hydroxypyrimidine
7.7521	0.220	4(1H)-Pyrimidinone, 6-hydroxy-
7.8174	0.170	2-Methylthiomethyl-4-(5-methylthiomethyl-2-tetrahydrothienyl)thietane
8.0014	0.237	Ethanamine, N-ethyl-N-nitroso-
8.0548	0.216	4(1H)-Pyridinone, 2,3-dihydro-1-methyl-
8.1260	0.659	4H-Pyran-4-one, 2,3-dihydro-3,5-dihydroxy-6-methyl-
8.2981	1.014	Semioxamazide
8.4109	0.317	(2-Benzenesulfonylamino-4-methylthiazol-5-yl)carbamic acid 2-dimethylaminoethyl ester
8.4822	0.263	Aziridine, 2,2,3,3-tetramethyl-
8.5653	0.332	2-Pyrrolidinecarboxamide, 5-oxo-, (S)-
8.6424	0.151	Piperidine, 3-methyl-
8.6958	0.315	1-Nonanamine
8.7492	0.627	Decane, 1-iodo-
9.0223	0.330	4-Acetamidobutyric acid
9.2181	1.058	2-Propanone, dimethylhydrazone
9.3072	0.625	Heptanal
9.3962	0.405	Deoxyspergualin
9.5505	0.227	Valeraldehyde, dimethylhydrazone
9.6277	1.309	Benzeneacetic acid
9.7820	0.763	1-Propene, 3-methoxy-2-methyl-
9.8770	0.424	o-(iso-Pentyl) S-(2-diethylaminoethyl) ethylphosphonothiolate
9.9600	0.348	Isosorbide
10.0313	0.505	Acetamide, N,N-bis(1-methylethyl)-
10.2271	1.452	Pentanal, 2-methylene-, 2-(1-methylethyl)hydrazone
10.3162	0.635	2,5-Diethylphenol

10.4111	0.392	1,3,5-Triazine, hexahydro-1,3,5-trimethyl-
10.5180	0.880	Phenol, 3-methyl-5-(1-methylethyl)-, methylcarbamate
10.5714	0.562	1H-Imidazole-4-methanol, 5-methyl-
10.6485	0.612	1,1'-Methylenebis(3-methylpiperidine)
10.7851	0.737	Oxazolidine, 2-isopropyl-2-methyl-
10.8444	0.578	1,3:2,4-Di-O-methylene-dl-xylitol
10.9334	1.460	Glycerol 1,2-diacetate
11.0284	1.532	Pyridine, 3-(1-methyl-2-pyrrolidinyl)-, (S)-
11.3192	1.130	L-Glutamic acid
11.4320	0.766	N-[4-Aminobutyl]aziridine
11.4914	1.024	Propane, 2-(ethenyloxy)-
11.6160	0.825	Prilocaine
11.7881	0.363	Oxazolidine, 2-ethyl-2-methyl-
12.5182	7.908	N1-(4-hydroxybutyl)-N3-methylguanidine acetate
12.5478	1.315	N1-(4-hydroxybutyl)-N3-methylguanidine acetate
12.6606	10.512	N1-(4-hydroxybutyl)-N3-methylguanidine acetate
13.0167	1.516	Thiourea, diethylaminomethyl
13.1414	0.464	.beta.-D-Glucopyranose, 1,6-anhydro-
13.2838	0.664	Piperazine
13.4678	0.474	Piperazine
13.5272	0.436	2-Naphthalenamine
13.7646	0.769	Cyclododecane
13.9248	0.756	3-Methyl-4-phenyl-1H-pyrrole
13.9723	0.296	4(1H)-Pyrimidinone, 6-hydroxy-
14.5362	2.509	2R,3S-9-[1,3,4-Trihydroxy-2-butoxymethyl]guanine
14.6845	2.366	2R,3S-9-[1,3,4-Trihydroxy-2-butoxymethyl]guanine
14.7498	2.417	2R,3S-9-[1,3,4-Trihydroxy-2-butoxymethyl]guanine
14.8863	0.473	2R,3S-9-[1,3,4-Trihydroxy-2-butoxymethyl]guanine
14.9398	1.015	N-Methoxymethyl-N-methylacetamide
15.2899	1.793	2-(2-Hydroxyethyl)piperidine
15.3730	1.291	1-Aza-8-oxabicyclo[4.3.0]non-3-en-2-one, 5-hydroxy-9,9-dimethyl-
15.6105	0.407	Pyrrolizin-1,7-dione-6-carboxylic acid, methyl(ester)
15.6461	0.714	Pterin-6-carboxylic acid
15.9250	1.934	Pyrrolo[1,2-a]pyrazine-1,4-dione, hexahydro-
16.1387	0.828	3-Piperidinemethanol
16.5126	1.222	l-Proline, N-allyloxycarbonyl-, heptadecyl ester
16.7678	0.394	2-Hydroxy-3,5,5-trimethyl-cyclohex-2-enone
17.1240	0.147	1,3-Benzenediol, 5-pentyl-
17.4979	0.545	Phenol, 3,4-dimethoxy-
17.5572	0.249	Pyrrolo[1,2-a]pyrazine-1,4-dione, hexahydro-3-(2-methylpropyl)-
17.6641	0.744	Pyrrolo[1,2-a]pyrazine-1,4-dione, hexahydro-3-(2-methylpropyl)-
17.7531	0.242	2,5-Cyclohexadien-1-one, 3,5-dihydroxy-4,4-dimethyl-
17.7946	0.895	1,2-Cyclopentanedione, 3,3,5,5-tetramethyl-
17.8777	0.566	n-Hexadecanoic acid
18.0736	0.163	Acetamide, 2-(adamantan-1-yl)-N-(1-adamantan-1-ylethyl)-
18.2101	0.336	9H-Pyrido[3,4-b]indole
19.3022	0.136	1,3-Propanediamine, N,N'-bis(3-aminopropyl)-
19.3972	0.114	Thiazolo[4,5-f]quinoline, 7-methyl-
19.5574	0.377	9-Octadecenoic acid, (E)-
19.7533	0.338	Octadecanoic acid
20.0323	0.113	Cyclohexanecarboxamide, N-(4-morpholylcarbonyl)-
20.1272	0.239	Terbutaline
20.5071	0.171	Formic acid, (2-fluoro-5-nitrophenyl)methyl ester
20.7564	0.775	1-Piperidinyloxy, 4-amino-2,2,6,6-tetramethyl-
21.3796	0.372	Ergotaman-3',6',18-trione, 9,10-dihydro-12'-hydroxy-2'-methyl-5'-(phenylmethyl)-, (5'.alpha.,10.alpha.)-
21.4805	0.173	Butyl 9-tetradecenoate

21.7416	0.700	Pyrrolo[1,2-a]pyrazine-1,4-dione, hexahydro-3-(phenylmethyl)-
24.1751	0.214	Formic acid, (4-fluoro-3-nitrophenyl)methyl ester
24.2523	0.281	Benzene, 1-methoxy-3-(methylthio)-
24.4956	0.654	l-Proline, N-allyloxycarbonyl-, propyl ester

Table S2. Chemical species identified by GC-MS in *S. rochei* DSM 41729 extract.

RT (min)	Area (%)	Assignment
3.2945	0.337	2-Propanol, 1-amino-
3.3657	0.459	N,N-Dimethylaminoethanol
3.5734	0.361	Ethyl 2,5,8,11,14-pentaoxahexadecan-16-oate
3.6387	4.057	2,3-Butanediol, [R-(R*,R*)]-
3.7159	1.983	2,3-Butanediol, [R-(R*,R*)]-
4.0957	0.258	Butanoic acid, 3-methyl-
4.1848	0.113	Butanoic acid, 2-methyl-
4.3510	0.300	1-Heptene, 5-methyl-
4.4697	0.204	3-Pyridinamine, 2-methyl-
4.9801	0.385	Butyrolactone
5.0395	0.134	Hydrazine, 1-methyl-1-(2-propynyl)-
5.0988	0.196	3H-Pyrazol-3-one, 1,2-dihydro-5-methyl-
5.3125	0.195	1,3-Butanediol, (S)-
5.6211	0.602	Benzenebutanoic acid, 3,4-dimethyl-.gamma.-oxo-
5.9535	3.590	1,1-Bis[aziridyltrimethylamine]
6.0544	0.736	Dimethylaminomethyl-isopropyl-sulfide
6.1078	0.903	N,N-Dimethylglycine
6.1969	1.553	N,N-Dimethylglycine
6.3927	1.641	N,N-Dimethylglycine
6.4817	3.237	Benzeneacetaldehyde
6.5945	1.422	Carbamic acid, 2-(dimethylamino)ethyl ester
6.8675	0.397	Oxirane, 2,3-diethyl-
6.9328	0.347	Butanedioic acid, 2,3-bis[(dimethylamino)methyl]-, diethyl ester
7.0575	0.654	2-Pyrrolidinone
7.1109	0.194	Butanenitrile, 4-(dimethylamino)-
7.1643	0.205	Butanenitrile, 4-(dimethylamino)-
7.2237	0.234	Inosine
7.2593	0.211	1,3-Propanediamine, N,N,N',N'-tetramethyl-
7.3127	0.202	N-[3-[N-Aziridyl]propylidene]-3-dimethylaminopropylamine
7.3720	0.339	But-3-enyl (E)-2-methylbut-2-enoate
7.4314	0.350	N,N-Dimethylacetamide
7.5916	0.146	1,2-Ethanediamine, N,N'-dimethyl-N-[2-(methylamino)ethyl]-
7.6985	0.391	4,5-Diamino-6-hydroxypyrimidine
7.8231	0.290	Piperazine, 1-[2-(2,5-dimethyl-1H-pyrrol-1-yl)ethyl]-
8.0130	0.195	Ethyldiethanolamine
8.0724	0.225	4(1H)-Pyridinone, 2,3-dihydro-1-methyl-
8.1377	0.498	4H-Pyran-4-one, 2,3-dihydro-3,5-dihydroxy-6-methyl-
8.2920	0.856	2-Phenylpropenal
8.4226	0.539	Dehydromevalonic lactone
8.4998	0.349	N,N-Dimethylacetoacetamide
8.5769	0.339	N,N-Dimethylacetoacetamide
8.6837	0.310	2-Piperidinone
8.7550	0.379	Thiazole
8.9805	0.215	N-Methoxymethyl-N-methylacetamide
9.0280	0.119	1,3-Dioxane-2-propanol, 2-methyl-
9.0695	0.238	2-Propenal, 3-(dimethylamino)-3-ethoxy-
9.1586	0.258	Benzenemethanamine, N-methyl-
9.3129	0.405	Pentanoic acid, 2,2-dimethyl-, methyl ester

9.3841	0.394	1H-Imidazole-4-carboxamide, 5-amino-2-methyl-
9.5503	0.229	5-Aminohexanoic acid
9.6334	1.057	Benzeneacetic acid
9.7818	1.790	dl-Mevalonic acid lactone
9.9658	0.290	2-Propanamine, N-ethyl-
10.0251	0.408	Urea, N-tert-butyl-N',N'-dimethyl-
10.1379	0.291	Deoxyspergualin
10.1913	0.214	Isosorbide
10.2566	0.455	Indolizine
10.3516	0.416	Deoxyspergualin
10.4109	0.238	3-Hydroxy-N,N-dimethylpropanamide
10.4525	0.244	Urea, N-tert-butyl-N',N'-dimethyl-
10.5118	0.495	3,4-Diethylphenol
10.5712	0.513	N,N-diacetylnorpseudoephedrine
10.6542	0.558	2-(Diethylamino)-N-(9,10-dioxoanthracene-2-yl)-ethanamide
10.7848	1.220	3-Hydroxy-N,N-dimethylpropanamide
11.0223	1.241	Isosorbide
11.1944	0.477	D-Leucine
11.3249	1.508	DL-Proline, 5-oxo-, methyl ester
11.4793	0.740	D-Leucine
11.5149	0.529	Isosorbide
11.6514	1.121	Leucine
11.6929	0.852	2,5-Diisopropylpiperazine
11.7879	0.537	3-[N-[2-Diethylaminoethyl]-1-cyclohexenylamino]propylnitrile
11.8591	0.753	D-Leucine
11.9660	1.089	D-Leucine
12.0490	0.964	D-Leucine
12.0906	0.290	Leucine
12.1321	0.489	D-Leucine
12.4171	4.866	Di-[1,3,2]-oxazino[6,5-f:5',6'-H]quinoxaline, 2,3,4,5,6,7-hexahydro-3,6-bis[2-diethylaminoethyl]-10,11-diphenyl-
12.4883	4.212	Dianhydromannitol
13.0284	0.291	Piperazine, 2,5-dimethyl-
13.0759	0.271	Arginine
13.1412	0.744	Ethyl isopropylamino oximinoacetate
13.5269	0.811	Isoquinoline, 1-methyl-
13.6457	0.399	4-Piperidinemethanol, 1-methyl-
13.7584	0.583	Cyclohexanone, 5-methyl-2-(1-methylethyl)-, cis-
13.8415	0.248	2-Butenal, dimethylhydrazone
13.9246	0.481	3-Methyl-4-phenyl-1H-pyrrole
13.9662	0.512	4-(4-Methylphenyl)pyridine
14.3341	0.514	Pyrazine, 2,5-dimethyl-3-propyl-
14.5716	1.017	1H-1,3-Benzimidazole-1-acetonitrile, 2-(difluoromethyl)-
14.6606	0.828	4-Methyl-2,7-dioxa-tricyclo[4.4.0.0(3,8)]decane
14.7378	0.538	2-Amino-3-cyano-5-aldoximinopyrazine-1-oxide
14.9336	0.410	.alpha.-Pyrrolidone, 5-[3-hydroxybutyl]-
14.9870	0.467	Isoglutamine
15.1117	0.418	l-Pyrrolid-2-one, N-carboxyhydrazide
15.1770	1.527	2-Piperidinecarboxylic acid
15.3847	1.371	3-Pyrrolidin-2-yl-propionic acid
15.4797	0.387	5-Amino-7-hydroxytriazolo(1,5-a)-S-triazine
15.5390	0.329	Benzenepropanoic acid, .alpha.-(1-aminoethyl)-, [R-(R*,R*)]-
15.6399	0.826	Pyridine, 3-(1-methyl-2-pyrrolidinyl)-, 1-oxide, (S)-
15.8892	2.094	Pyrrolo[1,2-a]pyrazine-1,4-dione, hexahydro-
16.0257	0.271	L-Threonine, N-[(1H-purin-6-ylamino)carbonyl]-
16.1325	1.017	Adenine
16.5302	3.181	Pyrrolo[1,2-a]pyrazine-1,4-dione, hexahydro-3-(2-methylpropyl)-

16.7736	0.654	Phenol, 3,5-dimethoxy-
16.8923	0.181	Arginine
17.1297	0.213	Imidazo[4,5-e]-1,4-diazepin-8-one, 1,4,5,6,7,8-hexahydro-1,4-dimethyl-
17.2840	0.153	7,10-Epoxy-6H-azepino[1,2-e]purine-8,9-diol, 4-amino-7,8,9,10-tetrahydro-, [7R-(7.alpha.,8.alpha.,9.alpha.,10.alpha.)]-
17.5036	1.663	l-Leucine, N-cyclopropylcarbonyl-, hexadecyl ester
17.6045	0.257	Phenol, 3,5-dimethoxy-
17.6757	2.376	Pyrrolo[1,2-a]pyrazine-1,4-dione, hexahydro-3-(2-methylpropyl)-
17.7529	0.521	Pyrrolo[1,2-a]pyrazine-1,4-dione, hexahydro-3-(2-methylpropyl)-
17.7944	0.751	5,10-Diethoxy-2,3,7,8-tetrahydro-1H,6H-dipyrrolo[1,2-a:1',2'-d]pyrazine
17.8775	0.502	n-Hexadecanoic acid
18.1209	0.101	2-Amino-4-nitrophenol, N,O-bis(methyl)-
18.1862	0.313	9H-Pyrido[3,4-b]indole
18.3642	0.271	Cyclododecanol, 1-ethenyl-
18.5245	0.135	N-8-Guanidino-spermidine
18.5957	0.156	Phenol, 2,3-dimethyl-
18.7322	0.105	2-(2-Hydroxyethylamino)-1,4-naphthoquinone
19.3198	0.211	[2,7]Naphthyridine-1,3,6,8-tetraol
19.4504	0.132	Spiro[5.6]dodecane-1,7-dione
19.7531	0.279	Octadecanoic acid
20.0973	0.131	Propionylfilicinic acid
20.5009	0.500	Diethyldithiophosphinic acid
20.6196	0.223	5-Isopropylidene-3,3-dimethyl-dihydrofuran-2-one
20.7383	0.836	Hexamethylcyclohexane-1,3,5-trione
21.2963	0.304	[1-(4-Amino-furazan-3-yl)-5-methyl-1H-[1,2,3]triazol-4-yl]-pyrrolidin-1-yl-methanone
21.3734	0.896	Pyrrolo[1,2-a]pyrazine-1,4-dione, hexahydro-3-(phenylmethyl)-
21.7414	2.537	Pyrrolo[1,2-a]pyrazine-1,4-dione, hexahydro-3-(phenylmethyl)-
24.1986	1.683	l-Norvaline, n-propargyloxycarbonyl-, tetradecyl ester
24.5013	1.981	Sebacic acid, 2,6-dimethoxyphenyl octyl ester

Table S3. Incidence of *B. cinerea* on cv. 'Red Globe' and cv. 'Timpson' grapes after 1, 5, 10, and 15 days. The numbers represent the percentage of clusters having more than one berry infected with *B. cinerea*. Values are means ± s.d. of 25 clusters with four replicates. Different letters represent significant differences by Tukey HSD test at $P < 0.05$.

**Time (days)**	**cv. 'Timpson'**				**cv. 'Red Globe'**			
	**Negative Control**	**Positive Control**	**COS-ML (1000 μg·mL⁻¹)**	**COS-MR (750 μg·mL⁻¹)**	**Negative Control**	**Positive Control**	**COS-ML (1000 μg·mL⁻¹)**	**COS-MR (750 μg·mL⁻¹)**
1	0 ± 0^a	0 ± 0^a	0 ± 0^a	0 ± 0^a	0 ± 0^a	0 ± 0^a	0 ± 0^a	0 ± 0^a
5	0 ± 0^a	19 ± 2^b	0 ± 0^a	0 ± 0^a	0 ± 0^a	0 ± 0^a	0 ± 0^a	0 ± 0^a
10	0 ± 0^a	76 ± 4^c	0 ± 0^a	0 ± 0^a	0 ± 0^a	33 ± 6^b	15 ± 2^b	0 ± 0^a
15	0 ± 0^a	100 ± 0^d	0 ± 0^a	0 ± 0^a	0 ± 0^a	80 ± 4^c	20 ± 1^c	0 ± 0^a

COS-ML: chitosan oligomers-*S. lavendofoliae* DSM 40217 secondary metabolites of. COS-MR: chitosan oligomers-*S. rochei* DSM 41729 secondary metabolites.
